# Diagnostik und Therapie traumatischer Aortenverletzungen

**DOI:** 10.1007/s00113-021-01044-0

**Published:** 2021-07-12

**Authors:** R. M. Benz, V. Makaloski, M. Brönnimann, N. Mertineit, H. von Tengg-Kobligk

**Affiliations:** 1 Diagnostische, Interventionelle und Pädiatrische Radiologie, Inselspital, Universität Bern, Freiburgstr. 18, 3010 Bern, Schweiz; 2 Universitätsklinik für Herz- und Gefäßchirurgie, Inselspital, Universität Bern, Freiburgstr. 18, 3010 Bern, Schweiz

**Keywords:** Klassifikation, Computertomographische Angiographie, Thorakale endovaskuläre Aortenrekonstruktion, Offene Aortenchirurgie, Leitlinien, Classification, Angiography, computed tomography, Thoracic endovascular aortic repair, Open aortic surgery, Guidelines

## Abstract

**Hintergrund:**

Traumatische Aortenverletzungen (TAV) sind seltene Folgen von stumpfen Traumata, die eine hohe Mortalität und Morbidität aufweisen. Die schnelle und akkurate Diagnostik sowie die Wahl der korrekten Therapie sind für das Patientenüberleben elementar.

**Fragestellung:**

Bestimmung des aktuellen Standards der Abklärung von TAV im akuten Trauma-Setting und Evaluation der aktuellen Leitlinien zur Therapie.

**Material und Methode:**

Eine Literaturrecherche wurde durchgeführt, mit der Suche nach Publikationen, die die Abklärung und Diagnostik der TAV beschreiben. Außerdem wurden Leitlinien für die Behandlung und Nachsorge von TAV zusammengefasst.

**Ergebnisse:**

In der Literatur wird trotz geringer Spezifität eine konventionelle Thoraxröntgenaufnahme als Initialdiagnostik genannt. Es sollte primär, als Modalität der Wahl, zur Diagnostik und zur Therapiestratifizierung eine Computertomographie (CT) aufgrund der hohen Sensitivität und Spezifität nachfolgen. In allen Leitlinien ist die thorakale endovaskuläre Aortenrekonstruktion („thoracic endovascular aortic repair“, TEVAR) die Therapie der Wahl bei höhergradigen TAV (Grade II–IV) und hat die offene Chirurgie in dem meisten Fällen abgelöst.

**Schlussfolgerung:**

Nach einer kurzfristig erfolgten CT-Diagnostik und Einteilung wird die TEVAR der offenen Chirurgie bei therapiebedürftigen TAV vorgezogen.

## Abstract (en)

**Background:**

Traumatic aortic injuries (TAI) are rare injuries in blunt thoracic trauma, which have a high morbidity and mortality. Rapid and accurate diagnosis as well as the correct choice of treatment are elementary for patient survival.

**Objective:**

Determination of the current standards for diagnostics of TAI in the acute trauma setting and evaluation of the current guidelines for treatment.

**Material and methods:**

A literature search was carried out for articles describing diagnostics of TAI. Furthermore, the guidelines for treatment and follow-up of TAI were summarized.

**Results:**

Despite the low specificity conventional chest X‑ray is still named in the literature as initial diagnostic procedure. Primarily, computed tomography (CT) should follow as the method of choice for diagnostics and treatment stratification due to the high sensitivity and specificity. Thoracic endovascular aortic repair (TEVAR) is recommended by all guidelines as first line treatment of higher grades of TAI (grades II–IV) and has replaced open surgery in most cases.

**Conclusion:**

After rapid diagnosis and classification of TAI with CT, in most cases TEVAR has become the preferred treatment over open surgery.

Zwar stellen traumatische Aortenverletzungen (TAV) seltene Ereignisse dar, aber aufgrund der hohen, mit ihnen assoziierten Morbidität und Mortalität ist die schnelle und akkurate Diagnostik für die Prognose des Patienten ausschlaggebend. Neben den traditionell-chirurgischen Therapieverfahren, die ebenfalls mit einer entsprechenden Morbidität und Mortalität einhergehen, haben sich in den letzten Jahrzenten zunehmend minimal-invasive Vorgehensweisen als Erstlinientherapie durchgesetzt und wurden in den nordamerikanischen und europäischen Guidelines verankert [10, 12, 21, 24, 28, 37, 43, 47]**.**

## Hintergrund

Die Mehrzahl der TAV ist durch stumpfe Traumata im Rahmen von Verkehrsunfällen, die in Form einer Kombination von Zug und Scherverletzung auftreten, verursacht [38]. Hierbei kann sowohl die thorakale als auch die abdominale Aorta verletzt werden, allerdings tritt Letzteres selten auf [7, 39]. Aufgrund der überwiegenden Mehrheit der thorakalen Aortenverletzungen bilden diese den Schwerpunkt des vorliegenden Beitrags. Sowohl in der Aorta abdominalis als auch in der Aorta thoracica kommen die Verletzungen an Prädilektionsstellen vor, was für die Unterscheidung einer akuten Traumafolge von einem chronischen Geschehen, wie z. B. einer arteriopathischen Dissektion, relevant ist.

Die schnelle und akkurate Diagnostik stellt einen dringlichen Beitrag zur erfolgreichen Therapie dar

Ungefähr 80 % der Patienten mit TAV erreichen das Krankenhaus nicht lebend. Die Mortalität beträgt aber auch bei Patienten mit technisch erfolgreicher Therapie noch bis zu 30 % [19]. Diese Zahlen unterstreichen die Dringlichkeit einer schnellen und akkuraten Diagnostik. Abhängig vom Schweregrad und von der Gefäßanatomie können die TAV konservativ, chirurgisch oder endovaskulär behandelt werden [34, 38, 41, 47]. Die endovaskuläre Therapie ist auch im deutschsprachigen Raum als „thoracic endovascular aortic repair“ (TEVAR) bekannt. Die Wahl des richtigen Therapieverfahrens, der Zeitpunkt der Therapie, aber auch eine begleitende Antikoagulation sind Gegenstand einer interdisziplinären Entscheidung und individuell auf dem Boden von Patientenalter, Komorbidität, Begleitverletzungen und Zustand des Patienten zu festzulegen [30, 41, 47].

## Diagnostik

### Bildgebende Untersuchungen

Obwohl eine konventionelle Thoraxröntgenuntersuchung in vielen Zentren zum initialen Screening gehört, ist diese aufgrund der niedrigen Spezifität von etwa 10 % und der fehlenden direkten Darstellung der Gefäße zur Diagnostik und zur Einteilung der TAV nicht geeignet [7, 9, 12]. Befunde im konventionellen Röntgenbild sind eine Verbreiterung des Mediastinums, der paraspinalen und paratrachealen Zone, eine abnorme Aortenkontur, eine Kompression oder Verlagerung der Luftwege, ein Verlust des aortopulmonalen Fensters, ein großer linksseitiger Pleuraerguss oder eine Verbreiterung des linken supraapikalen Weichteilschattens [19].

Im Schockraum hat sich als Initialdiagnostik nach Traumata die bettseitig durchgeführte Extended Focused Assessment with Sonography for Trauma (EFAST) zunehmend durchgesetzt. Diese ist allerdings zur TAV-Beurteilung ebenfalls nicht geeignet [19].

Mithilfe der CTA können essenzielle und akut therapierelevante Verletzungen dargestellt werden

In den meisten Zentren wird nach einer globalen Initialbeurteilung kurzfristig eine Kontrastmittel(KM)-gestützte computertomographische Angiographie (CTA) durchgeführt. Gründe sind die Verfügbarkeit, die Geschwindigkeit der Untersuchung sowie die Spezifität (fast 100 %) und hohe Sensitivität (96 %) [42]. Die detaillierten Bildakquisitionsprotokolle variieren zwischen den Häusern und sind von den jeweiligen Gegebenheiten abhängig. Sie beinhalten aber meist eine native CT und eine arterielle KM-Phase sowie fakultativ eine portalvenöse und/oder eine KM-Spätphase. In der Basis geht es darum, essenzielle und akut therapierelevante Verletzungen wie Frakturen, Organ- und Gefäßverletzung sowie arterielle oder venöse Blutungen schnell und akkurat zu diagnostizieren und möglichst exakt zu lokalisieren.

### Bildbefunde

#### Native Computertomographie

**Figure 1:**
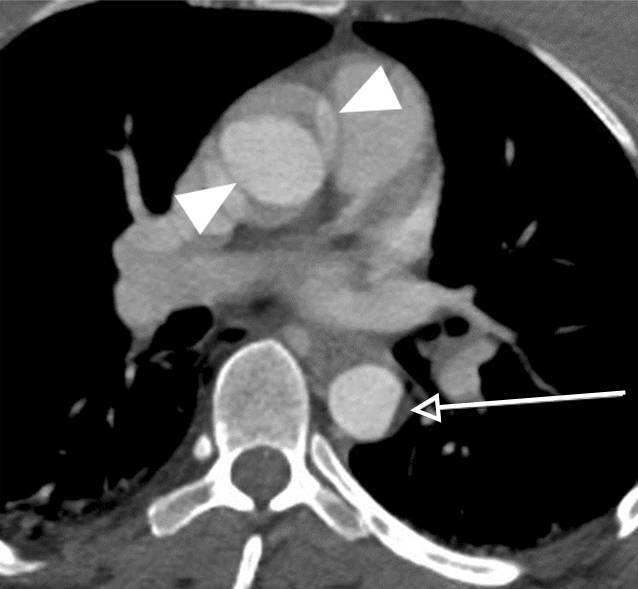

**Figure 2:**
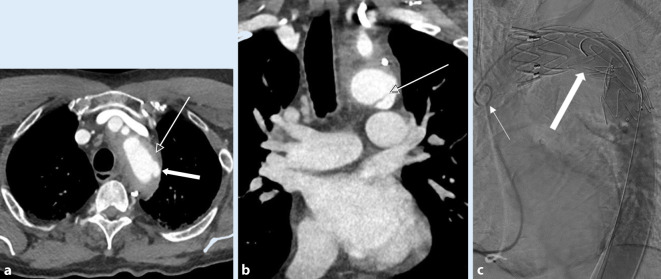

Indirekte Hinweise auf eine Aortenverletzung sind ein mediastinales Hämatom und/oder eine periaortale Fettgewebsimbibierung, v. a. wenn sie an den Prädilektionsstellen für TAV auftreten (Tab. 1; Abb. 1, 2b, 3a und 4a,b). Diese Prädilektionsstellen finden sich dort, wo die Aorta fixiert ist [7, 42].

**Table 1:** 

Thorax	Abdomen
Aortenisthmus (90 %)	A. mesenterica inferior (~33 %)
Aorta ascendens (5 %)	Nierenarterien (~25 %)
Hiatus des Diaphragmas (5 %)	Unterhalb der A. mesenterica inferior (~20 %)

**Figure 3:**
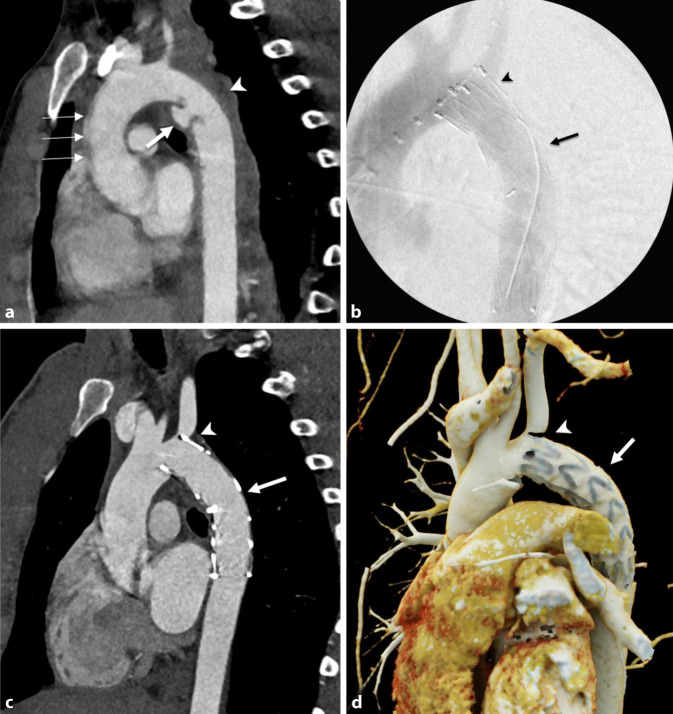

**Figure 4:**
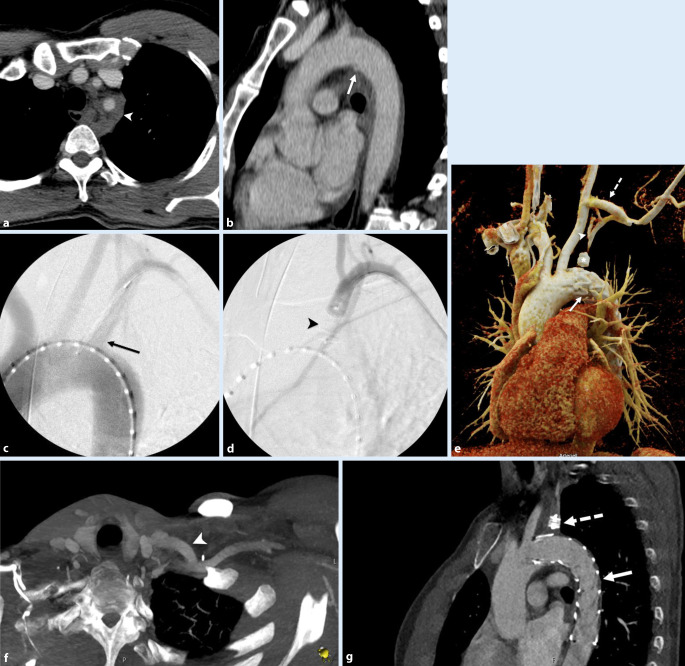

Auch andere Thoraxverletzungen können einen indirekten Hinweis auf eine TAV darstellen [31]. Abgesehen davon bietet bereits die native CT die Möglichkeit, ein akutes Wandhämatom von artheriosklerotisch-thrombotischen Wandauflagerungen zu unterscheiden [17]. Um diesen Effekt gut ausnutzen zu können, darf der Hämatokritwert des Patienten nicht zu niedrig sein (*Cave*: verdünntes Blut), und ein erfahrener Radiologe kennt auch weitere Unterscheidungskriterien auf Basis der KM-gestützten angiographischen Bildserie.

#### Computertomographische Angiographie

In der CTA, die mit jodhaltigem KM durchgeführt wird, wird die Aortenverletzung direkt dargestellt. Die Bildbefunde dienen zur Gradeinteilung der Aortenverletzung (Tab. 2), die Konsequenzen für die Art und den Zeitpunkt der Therapie hat.

**Table 2:** 

Grad	Aortenverletzung
I	Intimariss
II	Intramurales Hämatom
III	Pseudoaneurysma
IV	Ruptur

Die EKG-Triggerung ist zur Diagnostik von Läsionen der Aortenwurzel und Aorta ascendens essenziell

Ausdruck einer traumatischen Aortenverletzung sind die nachfolgend beschriebenen Befunde, wenn sie an den Prädilektionsstellen (Tab. 1) auftreten.

Ein intraluminaler Füllungsdefekt ist Ausdruck entweder eines Intimarisses (eher feinlinear; Abb. 2b) oder eines Thrombus (eher breitlinear; Abb. 1) oder von beidem. Eine abnorme Kontur der Aorta, im Sinne eines fokalen Kalibersprungs, ist entweder Zeichen eines muralen Hämatoms (Abb. 1) oder eines Pseudoaneurysmas (Abb. 2a, 3a und 4b). Im schwerwiegendsten Fall ist ein KM-Austritt erkennbar [7]. Die Differenzierung von Verkalkungen und KM ist mitunter schwierig, sodass eine vorangegangene native CT von Relevanz sein kann. Gegebenenfalls ist in der Spätphase eine Aussage über die Dynamik der Extravasation möglich, wobei die Therapie eines instabilen Patienten nicht aufgrund der bildgebenden Untersuchung verzögert werden sollte.

Einige nichtakute Bildbefunde können eine TAV vortäuschen. Dazu gehören ein Ductus diverticulum („ductus bump“), der sich v. a. durch einen breiten Hals gegen das eher schmalhalsige, traumatische Pseudoaneurysma abgrenzt. Im Fall eines Infundibulums, das sich als konische Ausstülpung darstellt, ist an dessen Spitze ein abgehendes Gefäß erkennbar. Auch Bewegungsartefakte können Dissektionen oder Konturunregelmäßigkeiten vortäuschen (Abb. 1 und 3a). Die indirekten Zeichen können zur Differenzierung hilfreich sein [15, 35]. Daher ist eine EKG-Triggerung zur Diagnostik Läsionen im Bereich der Aortenwurzel und der Aorta ascendens essenziell [46].

Ein transösophageales Echokardiogramm stellt eine Alternative dar; diese erfolgt bei hämodynamisch instabilen, ventilierten Patienten, die eine unmittelbare Diagnostik benötigen. Der Vorteil besteht darin, dass es am Patientenbett angefertigt werden kann [16]. Allerdings kann ein Segment der Aorta ascendens aufgrund einer Überlagerung der Trachea und des rechten Hauptbronchus nicht eingesehen werden [12].

Die katheterbasierte i.a.-Angiographie zur Diagnostik von Aortenverletzungen wird in der heutigen Zeit kaum noch durchgeführt [12].

## Therapie

### Thoracic endovascular aortic repair vs. offene Versorgung

Sind die Initialdiagnostik und Bilanzierung der Verletzungen abgeschlossen, geht es darum, die Therapien und deren Reihenfolge anhand der Relevanz zu triagieren. Die invasive Versorgung ist bei einer TAV der Grade II–IV indiziert. Die Typ-I-Verletzung wird konservativ und mit engmaschiger Überwachung behandelt [34]. Im Fall eines Progresses können leichtgradige TAV im Verlauf ebenfalls interventionsbedürftig werden.

Die TEVAR ist in den meisten Fällen die Therapie der Wahl. Auch wenn die Notwendigkeit einer Langzeitüberwachung nach TEVAR von einigen Autoren kritisch beurteilt wird [22], werden die Patienten meist über einen langen Zeitraum nachverfolgt, mit entsprechender Strahlenbelastung, die sich v. a. bei jungen Patienten akkumuliert [34, 49]. Ein Vorteil der offenen Versorgung ist das Wegfallen dieser Nachsorgeuntersuchungen. Allerdings birgt die chirurgische Therapie eine höhere Morbidität und Mortalität; dies wird im Abschn. Komplikationen detaillierter ausgeführt. Deswegen ist ein chirurgisches Vorgehen nur bei wenigen Patienten indiziert (Tab. 3) und wurde in den letzten Jahren zunehmend durch die in den meisten Guidelines empfohlene TEVAR ersetzt [10, 12, 21, 24, 28, 37, 43, 47].

**Table 3:** 

Endovaskuläre Versorgung	Chirurgische Versorgung
Multiple schwere Verletzungen Keine Beteiligung der Aorta ascendens Schwere rechtseitige Thorax- oder Lungenverletzung (Intoleranz einer Intubation) Geringe Lebenserwartung Multiple Komorbiditäten	Anatomische Gegebenheiten, die eine TEVAR ausschließen Beteiligung der Aorta ascendens Geplante offene Thoraxchirurgie zur Behandlung anderer Thoraxverletzungen

*TEVAR* „thoracic endovascular aortic repair“

Die überwiegende Zahl der Guidelines empfiehlt trotz Notwendigkeit der Langzeitüberwachung die TEVAR

Zur erfolgreichen Durchführung einer TEVAR sind gewisse anatomische Gegebenheiten Voraussetzung. Diese Kriterien müssen bei der Indikationsstellung und Planung der TEVAR berücksichtigt werden und sind in Infobox 1 zusammengefasst [20].

#### Infobox 1 Anatomische Kriterien für die Planung und Durchführung einer Thoracic endovascular aortic repair

- Aortendurchmesser ≥ 17,5 mm an der proximalen Landezone
- Proximale und distale Landezone ≥ 2 cm lang
- Geringe Kalzifikationen der Landezonen
- Fokale Läsion
- Keine signifikante Tortuosität der Aorta
- Zugangsgefäß mit adäquatem Durchmesser ohne relevante Stenosen oder Tortuosität
- Radius des Aortenbogens

Kotelis et al. haben basierend auf der bildgebenden Untersuchung verschiedene Aspekte einer Risikostratifizierung für die TEVAR vorgestellt, die im Einzelfall in Betracht gezogen werden können. Darunter fallen u. a. ein thrombogener und ein betont gotischer Aortenbogen [20, 25, 26].

### Thoracic endovascular aortic repair

#### Timing

Wenn die Indikation zur TEVAR gestellt ist, wird sie abhängig vom Grad der Verletzung sofort oder im Verlauf durchgeführt [28, 34]. Den besten Therapiezeitpunkt zu definieren, ist allerdings meist Teil eines komplexen und interdisziplinären Entscheidungsprozesses, v. a., wenn die TAV mit anderen schweren Verletzungen, wie z. B. des Gehirns, des Beckens oder der parenchymatösen Oberbauchorgane, einhergeht. Bei instabilem Patienten wird die TEVAR dringlich durchgeführt. Im Fall des hämodynamisch stabilen Patienten streitet sich die Literatur allerdings über den genauen Zeitpunkt. So empfiehlt z. B. die Society for Vascular Surgery in ihren Guidelines eine möglichst schnelle (< 24 h) Durchführung der TEVAR [28]. Dagegen empfiehlt die Eastern Association for the Surgery of Trauma (EAST) eine TEVAR im Verlauf (> 24 h; [13]). Eine 2021 publizierte Arbeit, die die Daten von 2821 Patienten aus der amerikanischen Traumadatenbank analysierte, berichtete jedoch ein deutlich besseres Ergebnis bei angepasstem Schweregrad im Fall einer TEVAR nach 24 h [1]. Auch die Frage nach einer prä- und/oder interinterventionellen Heparinisierung ist Gegenstand von individuellen Erwägungen [30].

#### Technik

Die TEVAR wird unter Vollnarkose durchgeführt. Ein transfemoraler Zugang erfolgt entweder via „cut down“ oder perkutan. Ein Pigtail-Katheter (Abb. 2c) wird im Aortenbogen für die Angiographie platziert (45°- bis 75°-linksanteriooblique Projektion, [20]). Der Endograft ist an den beiden Enden nicht bedeckt, wobei dieser Teil über den Abgang der A. subclavia abgesetzt werden kann (Abb. 3b–d). Falls eine Überdeckung der linken A. subclavia (ca. 30 %) nötig ist, wird im Fall einer dominanten linken Vertebralarterie oder eines Koronararterien-Bypass mithilfe der A. mammaria interna vorgängig in derselben Sitzung eine Revaskularisation mit einem Karotis-Subklavia-Bypass durchgeführt (Abb. 4c–g [20, 30]).

Die Größe des Endografts wird mit einem „oversizing“ von 15–20 % gewählt

Zusätzlich wird der Abgang der A. subclavia embolisiert, falls das Risiko eines Endoleak groß ist ([30]; Abb. 4d,e,g). Alternativ kann ein fenestrierter Graft in Erwägung gezogen werden, der in den letzten Jahren ebenfalls gute Resultate gezeigt hat [14, 27]. Der Endograft wird mit einem „oversizing“ von 15–20 % gewählt, das Oversizing kann aber im Notfall bis zu 30 % betragen, falls keine passende Größe vorhanden ist [30]. Eine Heparinisierung sollte im Einzelfall und in der Zusammenschau mit den Begleitverletzungen erwogen werden, ist aber bei der Implantation eines Karotis-Subklavia-Bypasses obligatorisch (mindestens 5000 IU). Der Endograft wird unter perpendikulärerer Fluoroskopie über einen supersteifen Draht an der proximalen Landezone positioniert. Nach angiographischer Kontrolle der Position wird dieser unter Apnoe freigesetzt [30, 34].

Falls in der Kontrollangiographie keine optimale Öffnung oder kein Kontakt zur Aortenwand ersichtlich ist, kann eine Angioplastie der proximalen und distalen Landezone durchgeführt werden [33].

Sollte die gesamte Aorta thoracica überstentet werden müssen, ist die Einlage einer spinalen Drainage zu erwägen, besonders wenn die distale Aorta betroffen ist. Dies verbessert die Durchblutung des Myelons durch Drucksenkung und verringert das Risiko für eine spinale Ischämie [33].

#### Nachsorge

Postintervention sollten die Patienten auf einer Intensivstation überwacht werden. Ein Monitoring der Vitalparameter und der neurologischen Funktion zum Ausschluss von zerebralen und spinalen Schlaganfällen sowie von Bein- und Mesenteriaischämien ist während 24 Stunden obligat [33]. Im Normalfall ist postinterventionell keine Heparinisierung notwendig.

Abhängig von Begleitverletzungen kann der Patient nach wenigen Tagen entlassen werden und normale physische Aktivität ist bereits nach wenigen Wochen wieder möglich [48].

Eine engmaschige Nachsorge mittels CTA (oder in speziellen Fällen mittels Magnet Resonanz Angiographie) nach 1, 6 und 12 Monaten und danach jährlich sollten im Verlauf durchgeführt werden, um ein Endoleak auszuschliessen [36, 48]. Im Falle eines Endoleaks sollte dieses entweder zeitnah angegangen werden (Typ I und III) oder engmaschiger kontrolliert werden (Typ II, IV und V) [4]. Ein positives Remodelling des Aortendurchmessers proportional zur Grösse des Oversizing des Stentgrafts ist dabei normal [2]. Bei jüngeren Patienten mit stabilem Bildbefund können die Kontrollintervalle im Verlauf verlängert werden, um eine exzessive Strahlenexposition zu vermeiden [22].

#### Resultate und Komplikationen

Im Vergleich mit der chirurgischen Versorgung ist das perioperative Ergebnis der TEVAR besser; die Mortalität nach TEVAR beträgt 7,5–12 % und nach chirurgischen Verfahren 19–23 % [10, 11, 23, 32, 44]. Mögliche Komplikationen können durch den Stent-Graft oder durch die Intervention bedingt sein. Komplikationen durch den Stent-Graft treten in 4 % der Fälle auf und beinhalten Endoleaks, Graft-Versagen (Verschluss, Diskonnexion der Komponenten etc.), technisches Versagen oder eine Verletzung der Iliakalgefäße [3, 5, 29]. Ein Graft-Versagen kann Folge einer falsch gewählten Stent-Größe sein und auf einer Unterschätzung des Aortendurchmessers im CT wegen einer vorliegenden Hypotonie beruhen. Der Versuch, ein Oversizing zu verhindern, kann in der Wahl eines zu kleinen Stent-Graft-Durchmessers mit ebenfalls konsekutivem Graft-Versagen münden. Meist können diese Komplikationen aber mithilfe einer endovaskulären Versorgung behoben werden [34]. Die präoperative Planung, empfohlen auf der Basis einer EKG-getriggerten Dünnschicht-CTA, ist ein essenzieller Beitrag zum erfolgreichen Ergebnis [18, 45]. Letztlich spart eine gute bildbasierte TEVAR-Planung auch Interventionszeit ein.

Das Risiko einer Myelonischämie ist bei der TEVAR im Vergleich zur Chirurgie (3 % vs.9 %, [34]) deutlich reduziert und konnte mit Einführung von durchblutungsfördernden Maßnahmen [11] in einigen Studien bis auf 0 % gesenkt werden [10, 11, 32]. Das Risiko eines Schlaganfalls beträgt etwa 1–10 % [8, 44] und steigt bei Überdeckung der linken A. subclavia von 2,7 auf 4,7 %, wie eine Studie zur elektiven TEVAR zeigte [6].

## Resümee

Die TAV ist eine seltene, aber umso häufiger letal verlaufende Verletzung, die unverzüglich diagnostiziert werden sollte, um die Mortalität zu senken. Die TEVAR als minimal-invasiver Eingriff wird in den meisten Guidelines, sofern nicht kontraindiziert, als Therapie der Wahl empfohlen.

## Fazit für die Praxis

- Bei einer traumatischen Aortenverletzung (TAV) ist meist die thorakale Aorta betroffen.
- Eine frühe, schnelle und akkurate Diagnostik mithilfe der EKG-getriggerten CT und computertomographischen Angiographie (CTA) trägt entscheidend zur Reduktion der Mortalität bei.
- Für die Diagnose und Einteilung ist sowohl eine native als auch eine Kontrastmittel(KM)-gestützte Darstellung der Gefäße mithilfe der CTA elementar.
- Bei TAV der Grade II–IV ist eine invasive Therapie indiziert.
- Die Therapie der Wahl stellt die minimal-invasive thorakale endovaskuläre Aortenrekonstruktion („thoracic endovascular aortic repair“, TEVAR) unter Einbezug von anatomisch relevanten Punkten dar.
- Eine CTA-basierte 3D-TEVAR-Planung ist ein essenzieller Baustein in der Versorgung.
- Im Fall eines hämodynamisch stabilen Patienten sollte die TEVAR elektiv nach 24 h durchgeführt werden.
- Morbidität und Mortalität sind geringer als nach chirurgischen Verfahren.
